# Hybrid GA-PSO Optimization for Controller Placement in Large-Scale Smart City IoT Networks

**DOI:** 10.3390/s25237119

**Published:** 2025-11-21

**Authors:** Sheeraz Ali Memon, Darius Andriukaitis, Dangirutis Navikas, Vytautas Markevičius, Algimantas Valinevičius, Mindaugas Žilys, Michal Prauzek, Jaromir Konecny, Zhixiong Li, Tomyslav Sledevič, Michal Frivaldsky, Dardan Klimenta

**Affiliations:** 1 Department of Electronics Engineering, Faculty of Electrical and Electronics Engineering, Kaunas University of Technology, Studentu g. 50-438, LT-51368 Kaunas, Lithuania; sheeraz.memon@ktu.edu (S.A.M.); dangirutis.navikas@ktu.lt (D.N.); vytautas.markevicius@ktu.lt (V.M.); algimantas.valinevicius@ktu.lt (A.V.); mindaugas.zilys@ktu.lt (M.Ž.); 2 Department of Cybernetics and Biomedical Engineering, VSB—Technical University of Ostrava, 17. listopadu 2172/15, 708 00 Ostrava-Poruba, Czech Republic; michal.prauzek@vsb.cz (M.P.); jaromir.konecny@vsb.cz (J.K.); 3 Faculty of Mechanical Engineering, Opole University of Technology, 45-758 Opole, Poland; z.li@po.edu.pl; 4 Department of Electronic Systems, Vilnius Gediminas Technical University, Saulėtekio Ave. 11, LT-10223 Vilnius, Lithuania; tomyslav.sledevic@vilniustech.lt; 5 Department of Mechatronics and Electronics, Faculty of Electrical Engineering and Information Technology, University of Zilina, 010 01 Žilina, Slovakia; michal.frivaldsky@uniza.sk; 6 Department of Power Engineering, Faculty of Technical Sciences, University of Priština in Kosovska Mitrovica, Kneza Miloša St. 7, RS-38220 Kosovska Mitrovica, Serbia; dardan.klimenta@pr.ac.rs

**Keywords:** controller placement, hybrid optimization, NB-IoT, GA-PSO, smart city

## Abstract

The Internet of Things (IoT) plays an important role in the development of smart cities. IoT forms a large network, and optimal controller placement plays a crucial role in ensuring network performance and resilience. This paper proposes a hybrid optimization approach that combines Genetic Algorithm (GA) and Particle Swarm Optimization (PSO) to strategically place controllers. Kaunas (Lithuania) was selected as a real-world smart city model. A large-scale Narrowband Internet of Things (NB-IoT) network with 2000 nodes was simulated, and 10 controllers were optimally placed in the network to minimize latency, balance load, enhance energy efficiency, and redundancy. The performance of the proposed hybrid GA-PSO algorithm was compared with random and K-Means clustering placements under three scenarios: normal operation, node failures, and traffic spikes. Simulation results demonstrate that the hybrid approach outperforms the other two methods in terms of load balancing, packet loss, energy efficiency, scalability, and redundancy. These findings highlight the robustness and effectiveness of the proposed hybrid algorithm in optimizing controller placement for smart city environments.

## 1. Introduction

The Internet of Things (IoT) plays an important role in the development of smart cities. The massive deployment of IoT devices introduced many challenges related to operation and management due to the heterogeneous and large number of connected devices. Among these challenges, the placement of network controllers is a major challenge [1] as the whole network depends on it. It plays a crucial role in the overall performance and resiliency of the network. To overcome this problem, Software-Defined Networking (SDN) provides a solution by decoupling the control and data planes, enabling centralized control and programmability. SDN Controller architecture can be broadly divided into centralized and distributed architectures [2]. In centralized SDN architecture, a single control plane manages the network traffic flow and resource allocation [3]. While distributed SDN architecture adopts a decentralized approach by spreading the control plane across multiple controllers, improving fault tolerance by having multiple controllers. It improves network resilience and scalability by distributing control tasks and reducing load on individual controllers [4]. Efficient controller placement directly impacts network performance metrics such as latency, load distribution, redundancy, energy consumption, and scalability. There are many standard protocols which are used for network connectivity in IoT; some of them are Bluetooth, 3GPP, LTE, 6LoWPAN, ZigBee, IEEE 802.15.4, NB-IoT, and 5G. These protocols define how the devices communicate with each other and cloud ensuring seamless data exchange [5]. If controllers are placed in random or sub-optimal locations, the network may suffer from high latency, controller overloading, poor redundancy, and reduced adaptability to changing conditions, particularly in a large-scale, distributed IoT environment. Finding an optimal solution for the number of controllers and their placement cannot be obtained in a short period of time. Once determined, the controller placement cannot easily be changed due to its complexity, making it essential to identify the best possible solution during the initial phase of design. Various algorithms and approaches have been reviewed to overcome these challenges and achieve an optimal solution [6].

The current research on controller placement in SDN-based IoT networks have mostly concentrated on static or small-scale topologies, whereas optimization is limited to a single performance parameter, such as latency, load balancing, or fault tolerance. Consequently, the research gap was addressed by a proposed hybrid Genetic Algorithm (GA) and Particle Swarm Optimization (PSO) framework that simultaneously optimizes numerous performance measures, specifically latency, load balancing, energy consumption, and fault tolerance, in a large-scale, real-world IoT environment. This study fills the gap by carrying out a realistic simulation of NB-IoT in Kaunas (Lithuania) city and evaluating performance under normal, failure, and traffic-spike conditions. A GA-PSO was proposed to address the mentioned limitation in IoT-based smart city network. The comprehensive methodology and simulation setup are explained in the Methodology section. The proposed method effectively combines the exploration capabilities of GA with the convergence properties of PSO [7]; it produces a robust and optimized distribution of controller positions across the city’s network. To validate the approach, a realistic geographic model of Kaunas, the second-largest city in Lithuania [8,9] along with NB-IoT range [10], nodes, and controllers, reflecting actual city conditions, was created. Whereas NB-IoT can support up to 50,000 devices [11]. The simulation uses three scenarios. These include normal operations, node failures, and traffic spikes so that it can thoroughly evaluate the robustness and adaptability of the hybrid algorithm. After that, its performance was compared against two methods, K-Means and random placement, using different metrics, including latency, load balance, redundancy, energy consumption, and scalability. The results demonstrate that the hybrid GA-PSO approach demonstrated superior performance compared to K-Means and random strategy based on defined metrics. The algorithm successfully minimizes latency and packet loss while improving redundancy and load distribution, which is desirable for large-scale smart city IoT networks.

Although hybrid GA-PSO algorithms have been studied before for controller placement, this has occurred mostly in small-scale network topologies and with few performance metrics. This study utilizes a hybrid GA-PSO approach within a realistic large-scale IoT framework, based on the actual geographical topology of Kaunas (Lithuania). The proposed approach utilizes a multi-objective fitness function that optimizes latency, load balancing, redundancy, fault tolerance, and scalability, while evaluating performance across various network situations, including regular operations, node failures, and traffic surges. This paper’s main contributions are summarized as follows:

- The proposed GA-PSO algorithm uses a multi-objective fitness function that reduces latency, controls load imbalance, reduces energy consumption, and enhances fault tolerance, which results in more reliable and energy-efficient IoT operations.
- A real city-based IoT network model of Kaunas (Lithuania) has been setup, comprising 2000 nodes, 10 controllers, and 2 communication ranges of 1000 m and 3000 m, to evaluate performance under valid geographic and density conditions.
- The algorithm’s performance is evaluated across three network scenarios: normal operation, random node failures, and traffic surges, to evaluate its robustness and adaptability in dynamic smart city environments.
- The comparative analysis with K-Means and random placement strategies illustrates the edge of the hybrid GA-PSO approach in terms of latency reduction, load balancing, energy efficiency, and redundancy.
- The paper provides outcomes that validate the proposed method’s applicability for large smart city IoT networks.

## 2. Related Work

The Controller Placement Problem (CPP) was first proposed by B. Heller et al. [12], who studied the influence of controller quantity and placement for the performance of SDN. This section combines various studies regarding controller placement and presents a brief overview of the research performed in this field.

### 2.1. Hybrid DEWO Algorithm for Controller Placement

Controller placement is one of the main issues in SDN-based IoT networks for smart cities, as it directly influences network performance metrics such as latency, load balancing, and fault tolerance. To address this, S. K. Keshari et al. [13] proposed a Hybrid Differential Evolution and Whale Optimization (DEWO) algorithm for optimizing controller placement. The algorithm was evaluated using MATLAB 2019b across three real-world topologies from the Internet Topology Zoo; these are TataNld, Deutsche, and Forthnet. Simulation parameters included a population size of 100, 200 maximum iterations, switch nodes varied from 50 to 150, and up to 40 controllers. The DEWO algorithm was compared with PSO and Firefly Algorithm (FFA) based on different metrics such as latency, fault tolerance, and link failure minimization. The results showed that DEWO outperformed both PSO and FFA, particularly in reducing end-to-end latency and minimizing link failures, thereby enhancing quality of service (QoS) in smart city environments. The results are summarized in Table 1.

Although DEWO demonstrated improved performance compared to PSO and FFA, still, some switch nodes were unevenly clustered near certain controllers. Moreover, simulations were conducted under static conditions, which may not fully reflect real-world dynamics.

### 2.2. GWOAP Algorithm for Load Management in SDN-IoT Networks

Load balancing is an important performance metric in SDN-based IoT networks for smart cities. It ensures optimal performance of controllers and minimizes communication cost. To address this, S. K. Keshari et al. [14] proposed a Grey Wolf Optimization Affinity Propagation (GWOAP) algorithm for intelligent controller placement and load distribution. The GWOAP was simulated in MATLAB 2019b using the OS3E topology. Consisting of 34 nodes (29 switches and 5 controllers) with 42 interconnecting links. The aim was to minimize total communication cost using a fitness function, with a weighting factor of 0.8 and flow rate set at 1 Kb/sec. The GWOAP was compared against different algorithms. These were GA, PSO, Genetic Algorithm with Affinity Propagation (GAAP), and Particle Swarm Optimization with Affinity Propagation (PSOAP). GWOAP results performed well compared to other methods in terms of balancing switch loads across controllers and reducing communication costs. It achieved optimal performance with 5 to 6 controllers and showed efficient execution times. Although the GWOAP algorithm achieved notable results, it still has several limitations. It was only validated through simulation with no real-world validation. Its scalability to large networks and adaptability to dynamic conditions were not evaluated. Additionally, the optimization was limited to communication cost, while other important metrics like energy efficiency, latency, and fault tolerance were not considered.

### 2.3. Multiple Distributed Controller Load Balancing (MDCLB) Algorithm for SDN-IoT Networks

Load distribution among controllers is critical in SDN-based IoT environments for smart cities, where performance metrics such as reliability and scalability are main concerns. To overcome this issue, H. Babbar et al. [15] proposed a Multiple Distributed Controller Load Balancing (MDCLB) algorithm. The algorithm aims to optimize CPU utilization across multiple controllers. It reduces packet drops due to load imbalance and minimizes network response times. Evaluations were performed using the Mininet emulator and Ryu controller in a large-scale SDN-IoT scenario with a linear topology via OpenFlow 1.3 protocol. The MDCLB algorithm was compared with other algorithms; these are Dynamic Load Balancing based on Nash Bargaining (DLBNB), Efficient Switch Migration Load Balancing (ESMLB), and Efficiency Aware Switch Migration (EASM). The authors used the iPerf test tool to simulate traffic loads and measure CPU utilization over a 0 to 20 s interval. CPU usage was measured before and after applying the load-balancing technique. The proposed MDCLB algorithm consistently performed well as compared to other algorithms by improving average CPU utilization and stabilizing performance across all controllers. The results are shown in Table 2.

MDCLB showed improved CPU utilization and consistent control-plane performance. It reduced latency and communication overhead compared to other algorithms. Despite its advantages, the MDCLB algorithm has several limitations. Its scalability to large networks is unclear. The algorithm used a fixed traffic load for load balancing, which raises concerns regarding dynamic traffic fluctuations. Additionally, evaluation was focused on CPU utilization, neglecting other important metrics like latency and scalability under real-world network environments.

### 2.4. Enhanced Sunflower Optimization (ESFO) and POCO Tool for Controller Placement in SD-IoT

Efficient controller placement is a main challenge in Software Defined Internet of Things (SD-IoT) networks. In this network, latency, fault tolerance, and load balancing significantly impact network performance. To address this, S. Hans et al. [16] proposed the Enhanced Sunflower Optimization (ESFO) algorithm, used in conjunction with the Pareto Optimal Controller Placement Tool (POCO) tool. This hybrid approach aims to determine optimal controller locations in wide area SD-IoT networks by minimizing delay and improving load balance across the control plane. The algorithm was validated through simulation using MATLAB and the results were compared against PSO, a hybrid SD method, and the PASIN algorithm. The results are summarized below in Table 3, which shows that the proposed algorithm significantly reduced average latency with two deployed controllers. These results highlight the importance of effective controller placement.

The ESFO algorithm, in conjunction with the POCO tool, effectively reduces latency. While the proposed approach has many advantages, it also has certain limitations. First, scalability for large-scale IoT networks was not covered, which increases uncertainties about performance in denser and more dynamic environments. Second, the evaluation was limited to simulation scenarios.

### 2.5. An Optimized Submodularity-Based Approach

A.K. Tran [17] proposed an optimization framework based on submodularity to address controller placement in large-scale IoT networks. The proposed approach focuses on multiple performance metrics, including execution time, number of controllers required, latency, and the impact of budget constraints. The proposed method was implemented in Python and evaluated based on execution time across different network sizes. The results demonstrated better results in terms of execution time, number of controllers, and network latency. Table 4 attached below shows the execution time in relation to network size.

For comparison, the Optimal method using the Gurobi solver was unable to process the 400-node network due to computational limitations, which highlights the scalability of the submodularity-based approach. Despite these advantages, the proposed method has several limitations. It may face challenges in highly dynamic environments where network conditions fluctuate rapidly. Additionally, scalability is unclear for very large IoT networks due to computational overhead, which was not fully examined in this study.

### 2.6. IoT-Aware VNF Placement (IVP) for Smart City Networks

Virtual Network Function (VNF) placement in IoT-based smart city networks faces significant challenges due to the dynamic and heterogeneous nature of IoT traffic. To address these, Y. Rafique et al. [18] introduced the IoT-aware VNF Placement (IVP) framework. It optimized placement under both static and dynamic traffic conditions. This approach is a multi-objective optimization approach to balance conflicting goals such as minimizing deployment costs while reducing network latency. The paper highlights the importance of Edge and Cloud infrastructures. The performance evaluation revealed that Mixed Integer Programming (MIP)-based algorithms suffer from significantly longer convergence times, ranging from 200 to 1000 times slower than heuristic alternatives, which makes them less suitable for real-time dynamic applications.

Case Study: Performance Evaluation of IVP Algorithms.

To validate the IVP framework, the authors presented a case study based on multiple objective smart city scenarios, including intelligent transport, public safety, smart energy, and healthcare applications. These use cases reflect a wide range of infrastructure and traffic requirements. Table 5 below shows the IoT traffic generated by different applications.

This highlights the importance of dynamic optimization algorithms that can adapt to diverse traffic patterns and service requirements.

### 2.7. PACSA-MSCP Algorithm

The Parallel Ant Colony leveraged by the Simulated Annealing for Multiple-Sink/Controller Placement (PACSA-MSCP) algorithm was introduced by H. R. Faragardi [19]. It achieved notable results in optimizing the placement of sink nodes and SDN controllers for industrial IoT networks. The algorithm achieves a deployment cost reduction of up to 19% compared to other methods. One of its main advantages is the reduction in execution time; for small-scale problems, PACSA-MSCP completed computation in approximately 4.2 min, whereas CPLEX took around 2 h. Similarly, for large-scale scenarios, PACSA-MSCP took 29.2 min compared to CPLEX’s nearly 10 h. Although the algorithm does not always find the exact optimal solution like CPLEX, it provides near-optimal results in at least one out of ten runs, with significantly less computational effort. Furthermore, comparing it with other heuristic algorithms such as Parallel Multi-Agent System (PMAS), GA, Simulated Annealing (SA), and Reactive Greedy Randomized Adaptive Search Procedure for Minimum Set Partitioning (R-GRASP-MSP), reveals that PACSA-MSCP consistently achieves better cost results on the small scale; it outperformed R-GRASP-MSP by 19% and for large scale by 12%. It has faster execution, which shows its computational efficiency and effectiveness. Table 6 presents a summary of the algorithm’s performance in comparison to CPLEX for both small- and large-scale problem scenarios.

The main strength of PACSA-MSCP is the reduction in deployment cost by merging colocated sinks and controllers. This technique works well, especially in small-scale scenarios and remains competitive in larger networks. In terms of contributions, PACSA-MSCP introduces a novel hybrid approach combining the Max-min Ant System with Simulated Annealing. This proposed approach significantly reduces the cost and improves timeliness and reliability for Industrial Internet of Things (IIoT) deployments. It handles scalability effectively and incorporates the practical benefit of colocating sinks and controllers to reduce infrastructure. Despite its advantages, it has some limitations. The complexity increases with more candidate locations, due to reduced efficiency. The proposed algorithm requires a minimum number of candidate sites to find feasible solutions. It does not address the overloading of nodes, which is important for maintaining real-time performance in industrial applications.

### 2.8. PHCPA Algorithm

N. Firouz et al. [20] proposed the PHCPA (proposed hybrid controller placement algorithm), which combines network partitioning with hybrid optimization techniques. Its performance was evaluated on six real-world SDN-based topologies from the Topology Zoo dataset. Simulation results demonstrated that PHCPA outperforms several meta-heuristic algorithms in minimizing network latency and improving network performance. The study also analyses the convergence rates, which show that PHCPA executes optimal solutions faster across different numbers of controllers. The hybridization of Manta-Ray Foraging Optimization (MRFO) and Salp Swarm Algorithm (SSA) enhances the search process, addressing slow convergence and local optima issues. The paper contributes by introducing a novel controller placement algorithm based on network partitioning and hybrid nature-inspired methods (MRFO and SSA). It proposes new discretization operators to adapt continuous algorithms to this discrete problem. Many experiments across six SDN topologies by varying numbers of controllers confirm the algorithm’s effectiveness and scalability. By emphasizing average switch-to-controller latency as the key performance metric, the study offers clear insights and outlines future research directions to advance controller placement strategies. However, the study has certain limitations, including reliance on older optimization algorithms such as PSO and FFA, which may reduce adaptability and efficiency in modern network environments. The study only focused on latency minimization while ignoring other important factors such as scalability, fault tolerance, and network adaptability. While PHCPA presents optimal approach for controller placement, these limitations should be carefully considered when applying it to real-world scenarios.

### 2.9. Multi-Objective Marine Predator Algorithm (MOMPA)

N. Firouz et al. [21] introduced a Multi-Objective Marine Predator Algorithm (MOMPA), to address controller placement problem in SDN. The proposed algorithm integrates the MOMPA algorithm with the Non-dominated Sorting Genetic Algorithm-II (NSGA-II) to enhance optimization performance. Proposed algorithm results demonstrated that MOMPA performed well compared to other algorithms by providing optimal solutions and improved computational efficiency. The use of mutation and crossover operators enables effective discretization of the algorithm, aligning it with the discrete nature of controller placement. The proposed algorithm focuses on multiple performance metrics, including installation cost, latency, load balancing, and imbalance, which demonstrates the proposed algorithm superiority across these metrics. This approach improves network performance by minimizing latency and balancing loads. The study also provides transparency in parameter settings to ensure reproducibility and outlines future research directions to further improve the algorithm’s applicability. Despite its advantages, it has several limitations. The hybrid nature of the algorithm increases computational complexity, which may pose challenges for deployment in large-scale and resource-constrained environments. The focus on latency and load balance overlooks other metrics such as security, fault tolerance, and scalability. Additionally, assumptions of ideal conditions, like no or single-link failures, do not fully represent real-world network scenarios.

### 2.10. Hybrid HSA-PSO Algorithm for Multi-Controller Placement in SDN

The optimal placement of controllers is essential for enhancing performance and fault tolerance in SDN. Radam et al. [22] introduced a hybrid HSA-PSO, combining the Harmonic Search Algorithm (HSA) and Particle Swarm Optimization (PSO) for multi-controller placement problem (MCPP) in SDN. The proposed model has three primary stages: (1) network construction, wherein the SDN topology is represented as an undirected graph; (2) optimal controller selection, in which the Firefly Algorithm (FA) determines the most appropriate controllers based on criteria such as modularity, API support, version, and compatibility; and (3) multi-controller placement optimization, where the hybrid HSA-PSO minimizes propagation latency and inter-controller communication delay. This hybrid methodology enables PSO to dynamically modify HSA parameters, hence improving global convergence and preventing premature local optima. The proposed model was analyzed using CloudSimSDN for propagation delay, round-trip time (RTT), reliability, throughput, and latency. The HSA-PSO algorithm was analyzed in comparison to Simulated Annealing Failure Foresight Capacitated CPP (SA-FFCCPP) and Garter Snake Optimization CPP (GSOCCPP). Simulation results showed that HSA-PSO performed better compared to GSOCCPP for delay metric by 50%, decreasing RTT to 7.3 ms in contrast to 14.7 ms, and reliability to 0.95. The hybrid model accelerated convergence and enhanced scalability. The proposed approach demonstrated significant results for delay reduction. It was analyzed solely through simulations. Future work may focus on scalability testing across various network topologies and real-world deployment situations to verify robustness and flexibility.

Table 7 summarizes all reviewed approaches, analyzing their performance metrics, advantages, and limitations, thereby providing a comprehensive overview of related studies in this field.

Overall, the algorithms analyzed above mainly focus on a single performance metric such as latency, load balancing, and packet loss, while neglecting other critical metrics essential for IoT networks, specifically energy efficiency, scalability, and redundancy. Moreover, these methodologies are validated only for small-scale networks, which do not represent the complex characteristics of large-scale IoT networks for smart cities. So, the present research indicates an important gap for simultaneously addressing several Quality of Service (QoS) metrics for large-scale IoT networks for smart cities. This study addresses the identified gap by proposing a hybrid GA-PSO controller placement method that focuses on improving multiple performance metric simultaneously in terms of latency, load balance, energy consumption, redundancy, and fault tolerance for large-scale IoT networks for smart cities.

## 3. Proposed Work

The GA-PSO was adopted for its effective optimization characteristics. GA applies crossover and mutation to introduce diversity into the population [23] but it typically suffers from slow convergence. GA has also been used for a variety of optimal placement problems across complex network systems [24]. In contrast, PSO, which is influenced by swarm intelligence, offers rapid convergence and computational efficiency via social and cognitive learning [25], but it is vulnerable to becoming trapped in local minima. Therefore, the proposed method combines GA and PSO for improved optimization performance. The proposed algorithm cooperatively integrates both approaches within a single optimization loop instead of sequentially. This allows GA to improve global exploration, while PSO improves convergence via local exploitation. The proposed approach maintains population variability and achieves improved results compared to random and K-Means independently for controller placement in large IoT networks. To evaluate proposed controller placement strategy, a large-scale IoT network simulation was created to realistically represent the city of Kaunas (Lithuania). The simulation was designed to reflect real-world conditions, making the results more practical and applicable. The implementation process consists of the following three main MATLAB scripts:

- init_network.m;
- hybrid_ga_pso_controller_placement.m;
- evaluate_performance.m.

**Figure 1:**
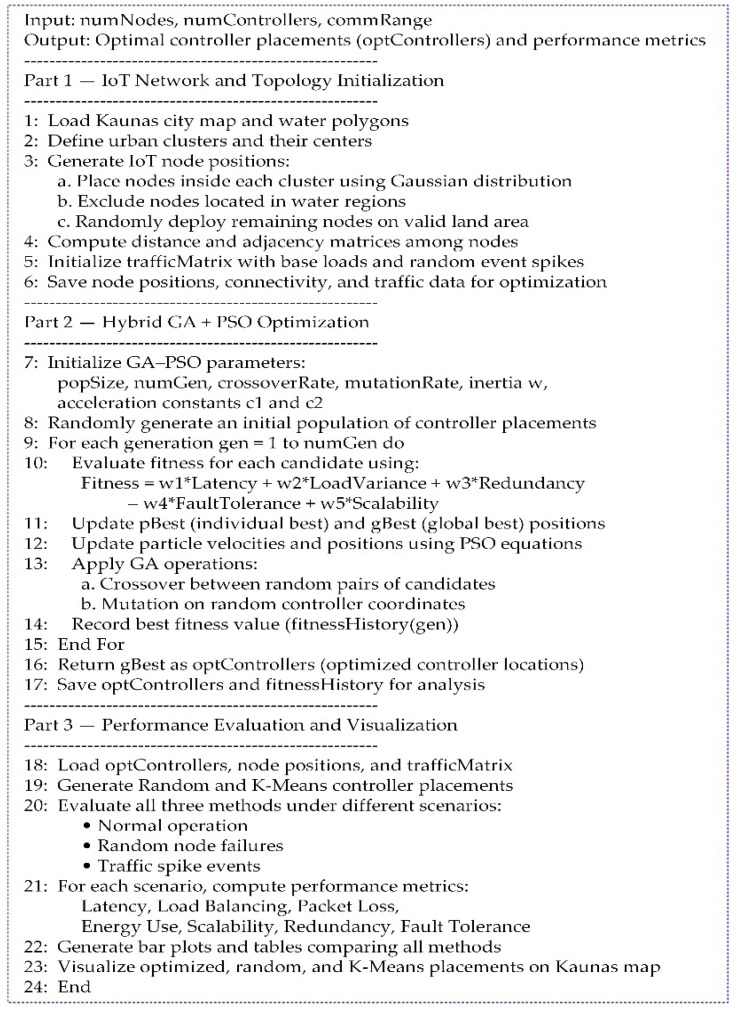
Pseudo-code (“*” denotes multiplication).

The pseudo-code in Figure 1 outlines the main steps of the MATLAB implementation.

**Figure 2:**
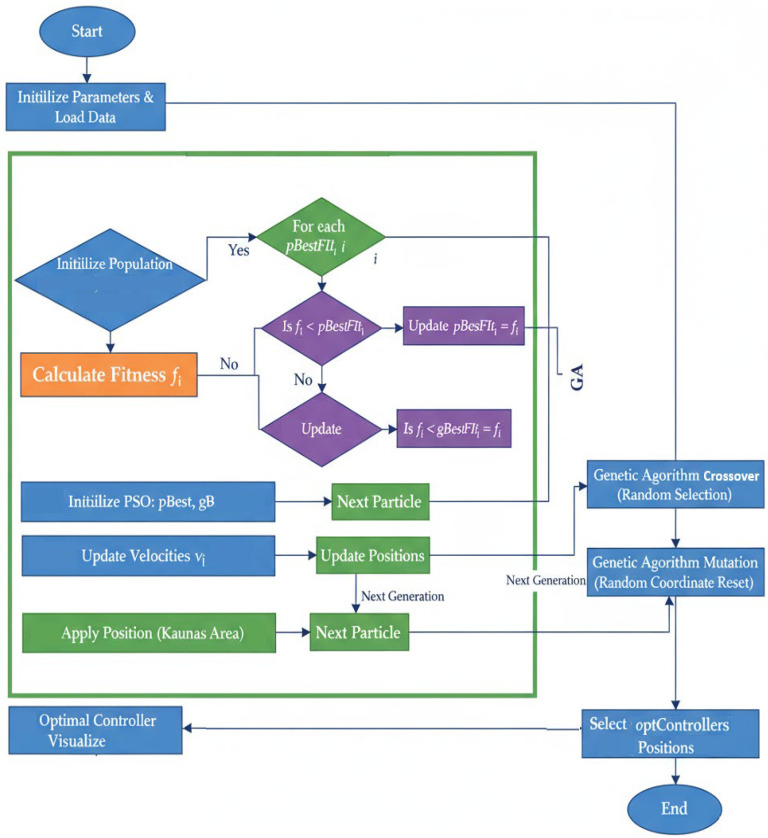
Flowchart of the Hybrid GA-PSO algorithm.

Figure 2 illustrates the workflow of the proposed hybrid GA-PSO technique. The method begins with data loading and the generation of a random population, succeeded by iterative fitness evaluation using PSO, exploration generated by GA through crossover and mutation, and convergence towards the optimal placement for the controller placement. Then, the optimized algorithm is compared against random and K-Means placement under normal, failure, and traffic-spike scenarios. A brief overview of each MATLAB script is provided below.

- init_network.m

In this study, a realistic simulation model was developed to represent the city of Kaunas (Lithuania) to support the investigation of optimized controller placement. The simulation includes 2000 IoT nodes deployed over a 15.35 km × 10.23 km area, reflecting the actual geographic dimensions of the Kaunas city located in Lithuania. Each node was analyzed for two communication ranges: 1000 m and 3000 m, with uplink traffic limited to 0.25 Mbps, using NB-IoT standards [10]. To emulate realistic urban deployment, a clustered spatial distribution model was applied with five high-density zones centered at coordinates [4000 3000; 12,000 8000; 8000 6000; 4000 4500; 4000 1500]. Each cluster has a radius of 2000 m, with nodes distributed using a Gaussian model [26]. Remaining nodes are randomly placed within the city area to simulate heterogeneous density. Connectivity was established by adjacency matrix linking nodes within 1000 m and 3000 m. For traffic modeling, nodes were assigned baseline loads between 0.1 and 1 Mbps. Additionally, 10% of nodes were designated as event-driven, simulating spikes of 2–4 Mbps, but all traffic was capped at 0.25 Mbps. To assess network robustness, a 5% node failure probability was introduced, and traffic spikes were simulated at 10% of the nodes. Then all generated data were saved in network_data.mat for use in subsequent optimization and performance evaluation stages.

- hybrid_ga_pso_controller_placement.m

To optimize controller placement using the hybrid GA-PSO approach, the algorithm was initialized with a population of 50 candidate solutions, where each individual represents coordinates for up to 10 controllers within the 15.35 km × 10.23 km area of Kaunas (Lithuania) city. The proposed algorithm uses a PSO framework, where each candidate’s position (controller locations) is iteratively updated over 100 iterations using inertia, cognitive, and social components. The control parameters for the hybrid GA-PSO optimization have been determined empirically through multiple initial trials. The population size was set at 50. The crossover and mutation rates have been set as 0.8 and 0.2, respectively. In the PSO part, the inertia weight (w) was set at 0.7, while acceleration coefficients (c_1 and c_2) were both set as 1.5, thus it is ensuring a balanced exploration–exploitation trade-off. Controller placement was evaluated using a custom fitness function that incorporates multiple criteria: traffic-weighted latency, controller load balance, redundancy, fault tolerance, and scalability. The multi-objective fitness function is expressed as

$$F=0.40\;L+0.25\;\sigma_{\mathrm{load}}+0.10\;R-0.15\;FT+0.10\;S$$

The optimization process evaluates each controller placement using a multi-objective fitness function which includes five essential metrics: latency, load balancing, redundancy, fault tolerance, and scalability. The fitness value in the MATLAB environment is computed as follows: f = 0.4 × totalLatency + 0.25 × loadVariance + 0.1 × avgRedundancy + 0.15 × faultTolerance + 0.1 × scalabilityScore. Total latency measures the traffic-weighted distance between IoT nodes and their authorized controllers, load variance calculates the difference in controller loads, and average redundancy indicates how much distance to the second-nearest controller. Fault tolerance denotes the proportion of nodes covered by multiple controllers within the communication range, whereas scalabilityScore indicates the location of controllers to avoid excessive clustering. The proposed method utilizes this fitness value to identify the optimum controller placement that improves latency, load distribution, and network resilience.

To ensure exploration and avoid premature convergence, crossover and mutation operations from GA are embedded into the PSO loop. Crossover combines controller positions between individuals, while mutation randomly alters individual controller coordinates with a small probability. The fitness function assigns each IoT node to its nearest controller, computes latency, load variance, average redundancy (second-nearest controller distance), fault tolerance (nodes within range of two or more controllers), and controller dispersion (to enhance scalability). After optimization, the optimal controller placement is saved into controller_results_kaunas.mat. A plot is then generated which shows the IoT node distribution along with the optimized controller locations. This visualization provides an overview of the controller deployment. The combination of GA and PSO is considered optimal as it maximizes the advantages of both algorithms: GA maintains population diversity and handles multi-objective optimization, whereas PSO ensures fast convergence. A previous study [7] has demonstrated that the combination of GA and PSO enhances accuracy and computational efficiency, therefore making it particularly suitable for large-scale IoT controller placement applications.

To evaluate the results of the proposed algorithm, performance metrics were calculated using the simulation. Latency was calculated as total latency is the sum of the product of minimum distances and the traffic vector. Here, minDists indicates the minimum Euclidean distance between each IoT node and its nearest controller, calculated by pdist2 (nodePositions, controllers), whereas trafficVector indicates the traffic load produced by each node. The multiplication of these numbers gives a traffic-weighted delay contribution per node, and the sum of them results in the total latency. The expression used for controller load balancing is expressed as loadVariance = var (controllerLoads), where controllerLoads represent the traffic loads allocated to each controller; a lower variance indicates more uniform utilization. Redundancy is defined as the mean of sortedDists (:, 2), which shows the average distance from each node to its second-nearest controller, indicating the availability of alternative pathways. Fault tolerance is defined as fault. Tolerance = multiReachNodes/n, which measures the ratio of nodes linked to a minimum of two controllers within the communication range, hence indicating the network’s robustness against controller failures. Scalability was determined using the formula scalabilityScore = 1/avgCtrlDist, representing the inverse of the average inter-controller distance, which prevents controller clustering and encourages extensive spatial coverage. In the end, energy efficiency was evaluated indirectly through reduced latency and communication distance.

- evaluate_performance.m

This script initiates by loading two MATLAB files: network_data.mat, which contains the node positions, traffic matrix, communication range, failure probabilities, traffic spike node information, and controller_results_kaunas.mat, which stores the optimized controller coordinates generated by the proposed hybrid GA combined with PSO (GA-PSO) method. The performance of the optimized controller placement is then evaluated across three different network scenarios:

- Normal Operation: In this scenario, no node failures occur. The script invokes the function evaluateMetrics using the optimized controllers, current node positions, and traffic matrix. This function computes main network performance metrics including latency, load balancing, packet loss, energy consumption, scalability, redundancy, and fault tolerance.
- Random Failures: To simulate network instability, a percentage of nodes are randomly designated as failed based on a predefined failure probability (failProb), which is 5%. The failed nodes are excluded from the performance evaluation. The metrics are recalculated considering only the active nodes, which reflect the network’s robustness under partial node failures.
- Traffic Spikes: To mimic real-world traffic surges, 10% of the nodes are randomly selected as spike nodes. Their traffic demand is doubled in the traffic matrix. The script evaluates the network metrics under this increased load, providing insight into the controller placement’s ability to handle sudden traffic spikes.

For evaluation and comparison purposes, two additional controller placement strategies were used; these were random placement and K-Means Placement [27]. The same set of performance metrics was computed for each placement strategy by calling evaluateMetrics. The script then creates a structured table that summarizes the results for the optimized, random, and K-Means controller placements, which helps in direct comparison of their results. In the last script, a results figure was produced with subplots displaying bar charts for each metric, accompanied by clear labels and grid lines to enhance interpretability.

## 4. Results and Discussion

The main objectives of this study are to find the optimal controller placement by reducing latency, achieving balanced load distribution across controllers, increasing energy efficiency, ensuring redundance, fault tolerance, and increasing network scalability within a large-scale IoT network. Each metric’s result demonstrates that the proposed algorithm achieved improved results compared to random and K-Means methods, starting with an analysis of the initial script.

- init_network.m

**Figure 3:**
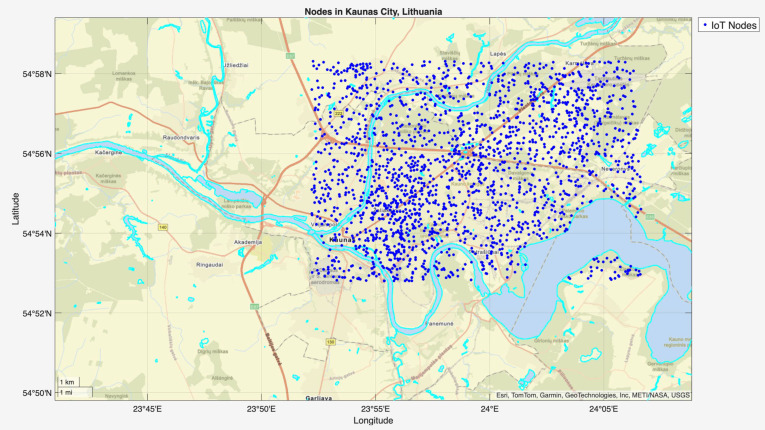
Nodes deployment in Kaunas (Lithuania) city topology.

To generate the simulation environment, the MATLAB script init_network.m was executed, which creates a realistic model of IoT-based smart city using Kaunas (Lithuania) city topology. Figure 3 illustrates the simulated IoT network topology for Kaunas (Lithuania) city generated by the init_network.m script. It shows 2000 nodes distributed over a 15.35 km × 10.23 km area, where 20% of the nodes are clustered around five urban centers [4000 3000; 12,000 8000; 8000 6000; 4000 4500; 4000 1500] and the remaining 80% nodes are scattered in less dense regions. Each node has been analyzed for two communication ranges: 1000 m and 3000 m. The connectivity and distance matrices were constructed to support routing and controller analysis. Traffic loads followed NB-IoT protocol, where most nodes produced low-rate data (0.1–0.25 Mbps), while 10% of the nodes were modeled as event-based, experiencing additional traffic spikes and 5% failure probability of nodes was also introduced to reflect real-world issues.

- hybrid_ga_pso_controller_placement.m

**Figure 4:**
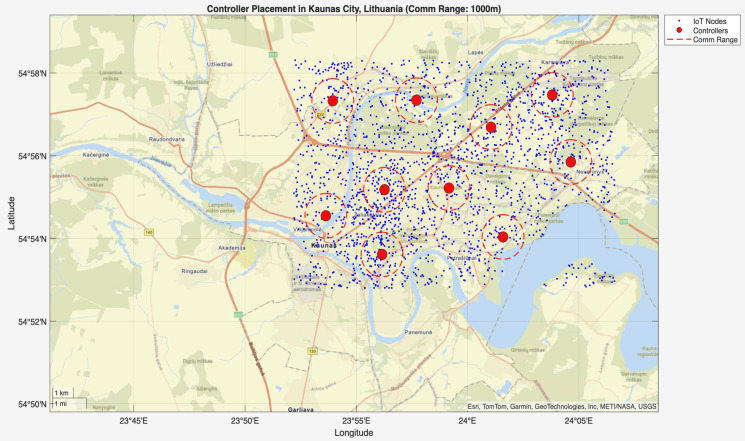
Optimal controller placement across Kaunas (Lithuania) city with a communication range of 1000 m.

**Figure 5:**
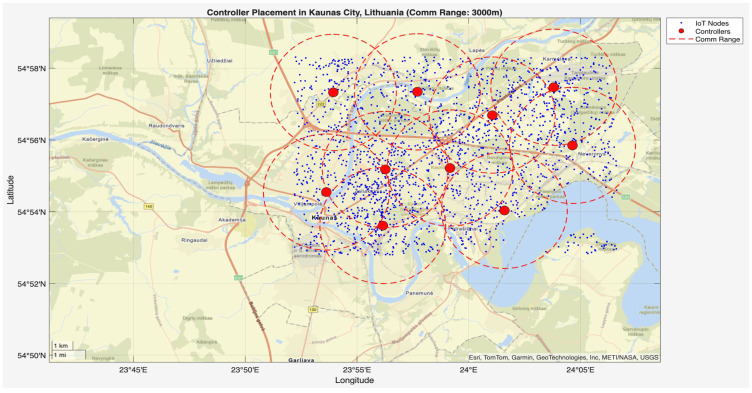
Optimal controller placement across Kaunas (Lithuania) city with a communication range of 3000 m.

In the second stage of the simulation, the script hybrid_ga_pso_controller_placement.m was executed following the initialization by init_network.m. This phase applied GA-PSO to determine the optimal controller placement across the Kaunas (Lithuania) city area. The algorithm used a population size of 50, 100 iterations, and optimized controller positions within a 15.35 km × 10.23 km area containing 2000 IoT nodes. Figure 4 and Figure 5 show the controller placement with communication ranges of 1000 m and 3000 m, respectively, where 10 controllers are optimally placed across the model city. Controllers are represented by red dots across the city, blue dots indicate the nodes, and the red circle shows the communication range between the controller and the nodes within it. Controllers were strategically placed in both high-density urban centers and peripheral regions, which reflects the multi-objective fitness function’s success in optimizing communication efficiency, redundancy, and scalability. These results confirm the robustness of the hybrid GA-PSO approach for practical controller placement in real-world smart city IoT networks.

- evaluate_performance.m

**Figure 6:**
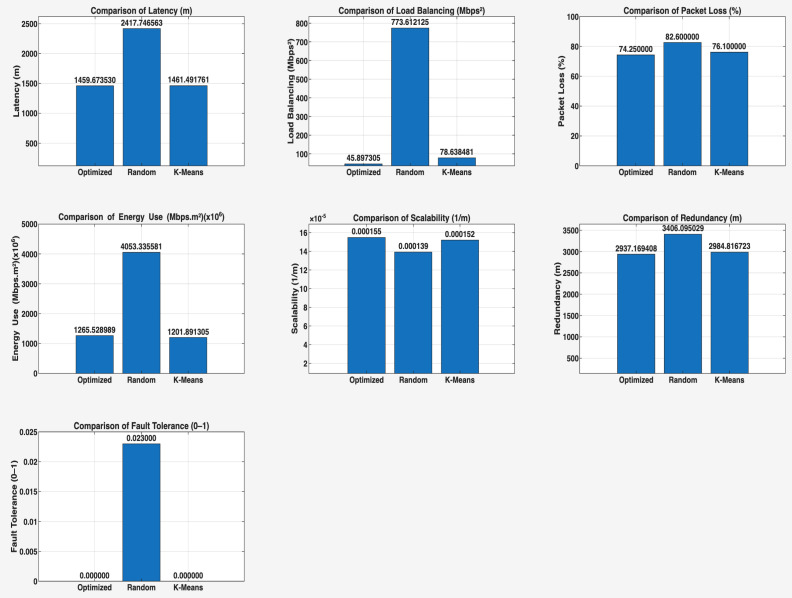
Comparison of the proposed algorithm with K-Means and random placement strategies for a comm. range of 1000 m.

**Figure 7:**
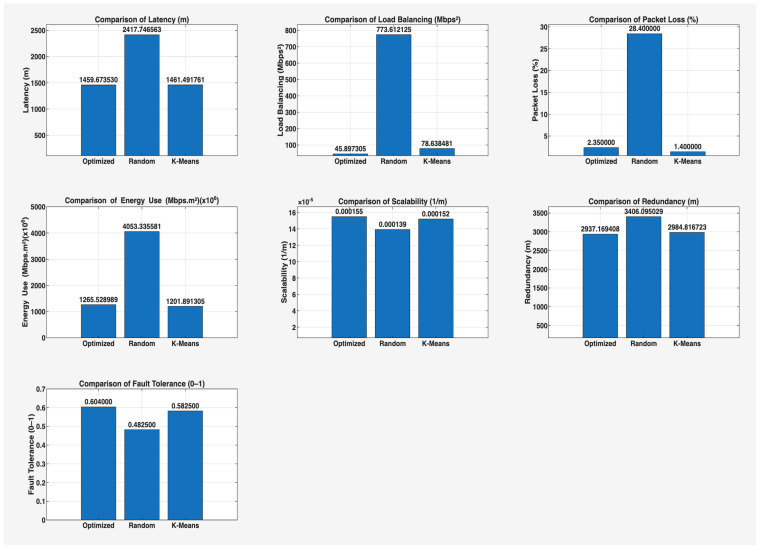
Comparison of the proposed algorithm with K-Means and random placement strategies for a comm. range of 3000 m.

**Figure 8:**
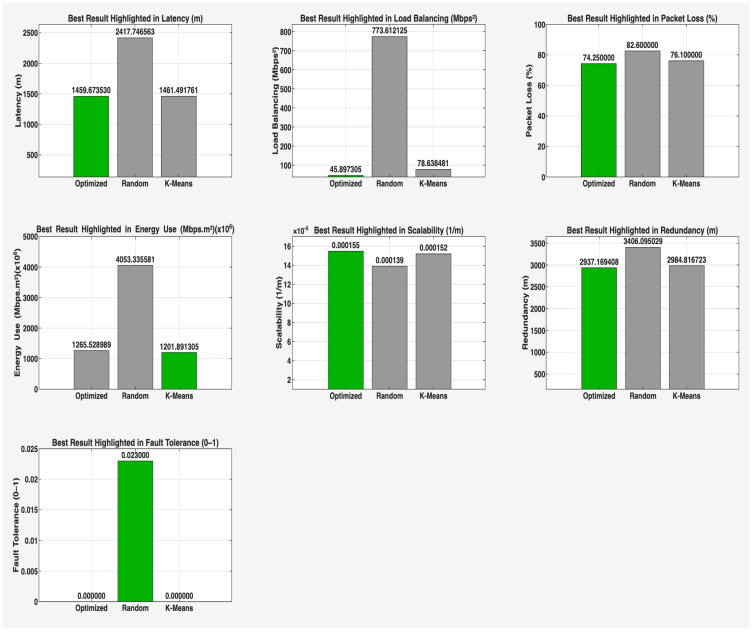
Highlight best-performing controller placement strategy in green color for a comm. range of 1000 m.

**Figure 9:**
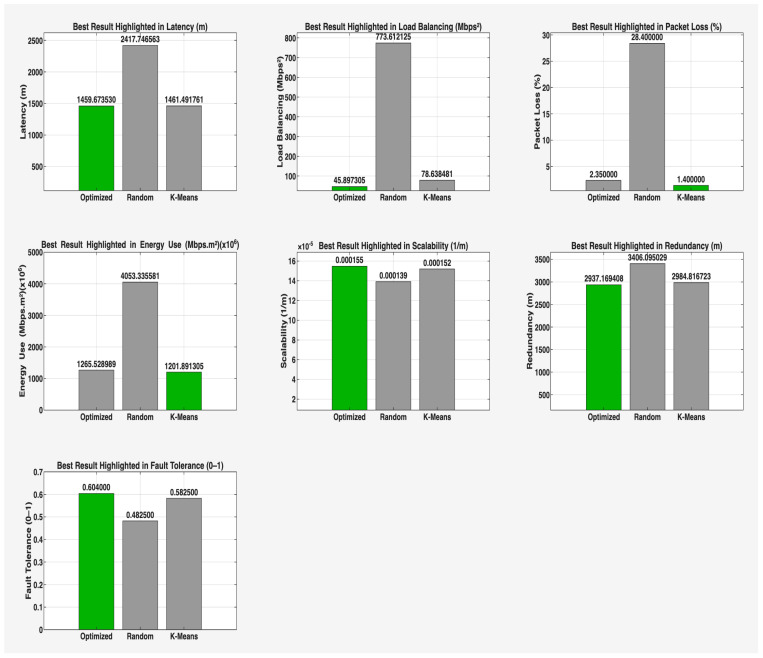
Highlight best-performing controller placement strategy in green color for a comm. range of 3000 m.

**Figure 10:**
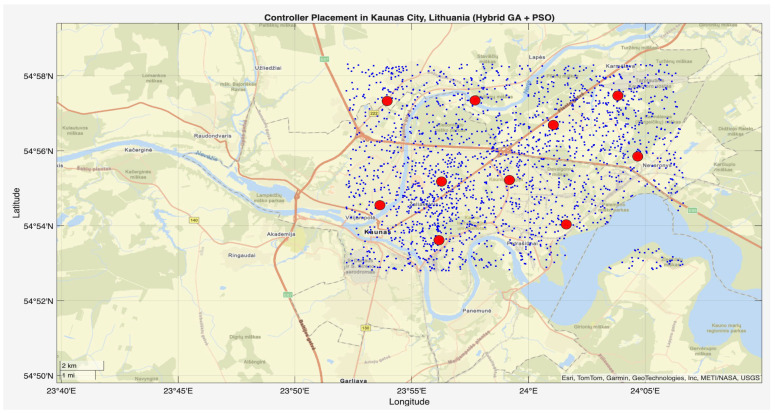
Optimized controller placement.

**Figure 11:**
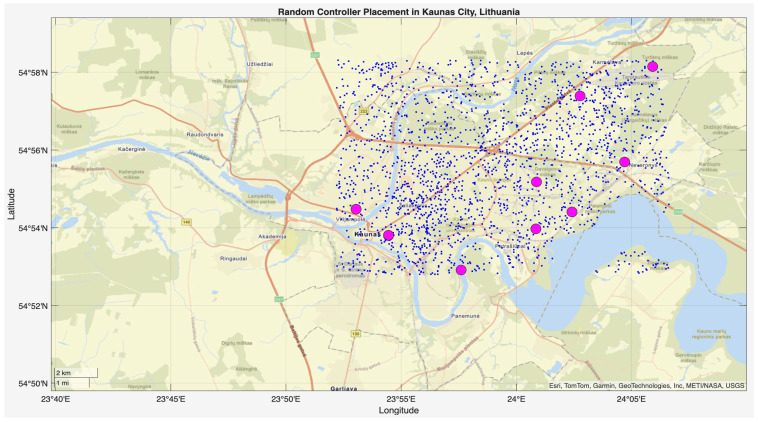
Random controller placement.

**Figure 12:**
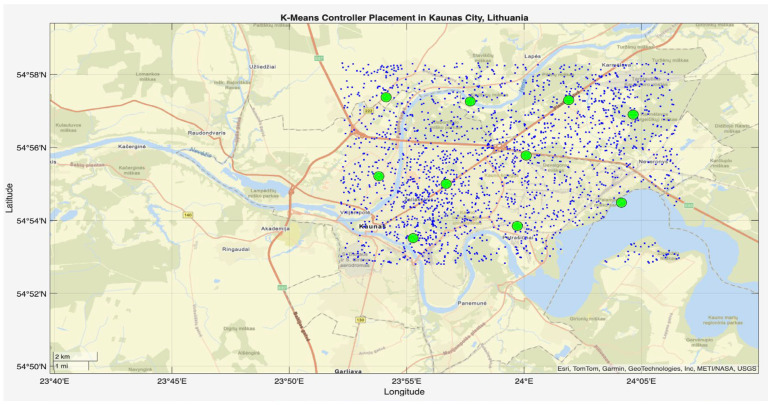
K-Means controller placement.

**Figure 13:**
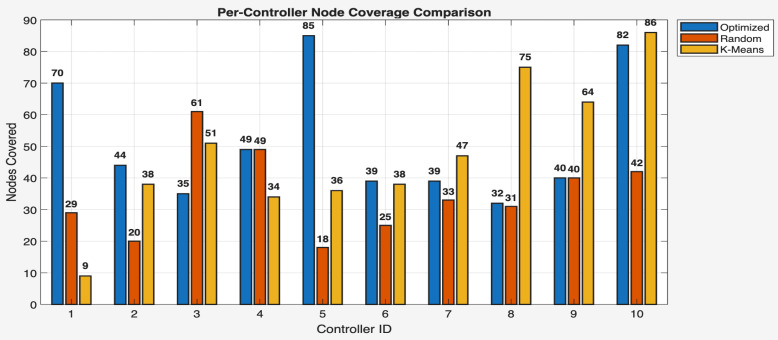
Nodes coverage per-controller comparison for optimized, random, and K-Means at 1000 m comm. range.

**Figure 14:**
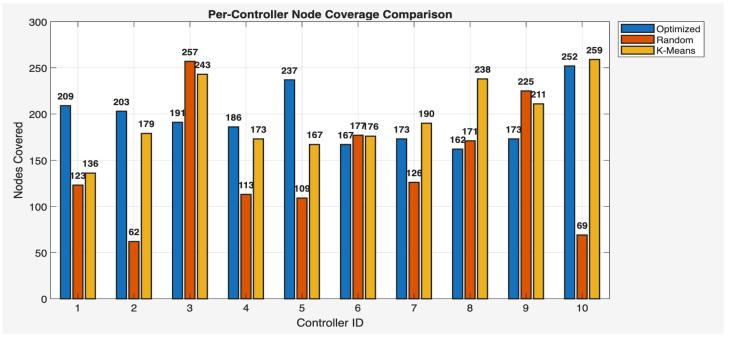
Nodes coverage per-controller comparison for optimized, random, and K-Means at 3000 m comm. range.

This phase of the study involved evaluating the performance of the proposed algorithm in large-scale IoT networks. The evaluation was carried out using the evaluate_performance.m script, which compared three different controller placement strategies: optimized hybrid GA-PSO, random, and K-Means clustering. The evaluation metrics included latency, load balancing, packet loss, energy consumption, scalability, redundancy, and fault tolerance. The results are shown in Figure 6 and Figure 7 for communication ranges of 1000 m and 3000 m, respectively, with the best results highlighted in green in Figure 8 and Figure 9. Figure 10 illustrates the optimized controller placement, Figure 11 shows the random controller placement, and Figure 12 presents the K-Means controller placement and their results are summarized in Table 8. Here is a summary of results in terms of latency, which measures the average delay between nodes and their assigned controllers: results for latency are identical for 1000 m and 3000 m communication range, the optimized algorithm achieved the low latency of 1459.67 m, which outperforms the K-Means method, which resulted 1461.49 m, while random placement achieved the high latency at 2417.74 m. Latency results align with the theoretical and practical results of Heller et al. [12]. Their study revealed that random controller placement normally results in average latencies 1.4×–1.7× times greater than those of optimal placements. Similarly, the proposed hybrid GA-PSO method achieves an approximate 39.6% reduction in latency compared to random placement, which is approximately identical with the improvement ratio observed in Heller’s study. Load balancing reflects variance in traffic load across controllers. All load balancing results are identical for both communication ranges. Optimized algorithm achieved a low variance of 45.89 Mbps^2^, compared to 78.63 Mbps^2^ for K-Means and 773.61 Mbps^2^ for random placement. This indicates the hybrid method effectively distributes the node load, reducing congestion and ensuring more stable network performance. Packet loss is percentage of nodes outside the communication range of controller; thus, the communication range affects the packet loss. The optimized method achieved a lower packet loss rate of 74.25%, compared with 76.10% for K-Means and 82.60% for random placement at the 1000 m range. While for the 3000 m range, K-Means performed slightly well, due to its centroid node-based clustering approach [28], which results in very few nodes becoming unreachable at the 3000 m range. Therefore, packet loss is slightly lower compared to the proposed algorithm. However, it lags in all other essential performance metrics such as latency, load balancing, and redundancy. The packet loss results at 3000 m range are 2.35% for optimized, for random 28.40%, and 1.40% for K-Means. Regarding energy consumption, which was modeled as proportional to the square of the distance between nodes and their assigned controllers, algorithms achieved identical results for both communication ranges. K-Means controller placement consumed the lowest energy by achieving 1201.89 Mbps.m^2^ (×$10^{6})$, followed by the optimized algorithm 1265.52 Mbps.m^2^ (×$10^{6})$, while random placement consumed 4053.33 Mbps.m^2^ (×$10^{6})$. K-Means performed slightly well for the energy consumption metric compared to the proposed algorithm, due to its centroid node-based clustering approach; K-Means placed the controller near to node clusters, which minimizes distances between nodes and the controller [28,29]. As a result, transmission power requirement is reduced. Therefore, the energy consumption is marginally lower compared to the proposed approach. However, this clustering base placement comes at the cost of poor performance in other performance metrics such as latency, load balancing, and redundancy. In terms of scalability, which was defined as the inverse of the average inter-controller distance, all algorithms obtained identical results for both ranges. The optimized approach achieved 0.000155 1/m, while random placements had the lower value of 0.000139 1/m, followed by K-Means’ 0.000152 1/m. This suggests that the optimized method has greater scalability as network size increases. Redundancy, measured as the average distance to the second-nearest controller, results are identical for both ranges; the optimized configuration achieved 2937.16 m, which is slightly lower than K-Means’ 2984.81 m and random placement with 3406.09 m. Lower redundancy distance means better backup of controller availability in case of failure, which is essential for resilient smart city applications. Finally, fault tolerance, defined as the proportion of nodes within the range of two or more controllers, results are different for both communication ranges: the optimized algorithm achieved a fault tolerance value 0.00 and 0.60, random placement achieved a 0.023 and 0.48, while K-Means achieved 0.00 and 0.58. Zero value here shows that it failed to provide overlapping coverage for any node. All performance metric findings are identical across both communication ranges, except for packet loss and fault tolerance, which exhibit different results. The communication range affects only these two metrics. Other performance parameters, such as latency, load variance, energy, redundancy, and scalability, are distance-based and independent of the communication range within this setup. Thus, increasing the range from 1000 m to 3000 m does not change nearest-controller allocations or node-controller distances; consequently, the results remain unchanged. However, the communication range influences node coverage. At 1000 m, the proposed hybrid GA-PSO covers 25.75% of nodes (515/2000), compared to 23.9% for K-Means (478/2000) and 17.4% for random (348/2000). At 3000 m, nodes coverage significantly increases: hybrid GA-PSO covers 97.65% (1953/2000) node, K-Means 98.6% (1972/2000) nodes, and random 71.6% (1432/2000) nodes. Although K-Means shows slightly higher coverage at 3000 m, the optimal placement ensures balance load per-controller (162–252 nodes) compared to random (62–257) and is identical to K-Means (167–259), and hence the proposed algorithm reduces overload risk and facilitates stable operation. Figure 13 and Figure 14 illustrate the per-controller node coverage for optimized (hybrid GA-PSO), random, and K-Means placements at communication ranges of 1000 m and 3000 m, respectively, while Table 9 provides the corresponding numerical data.

**Figure 15:**
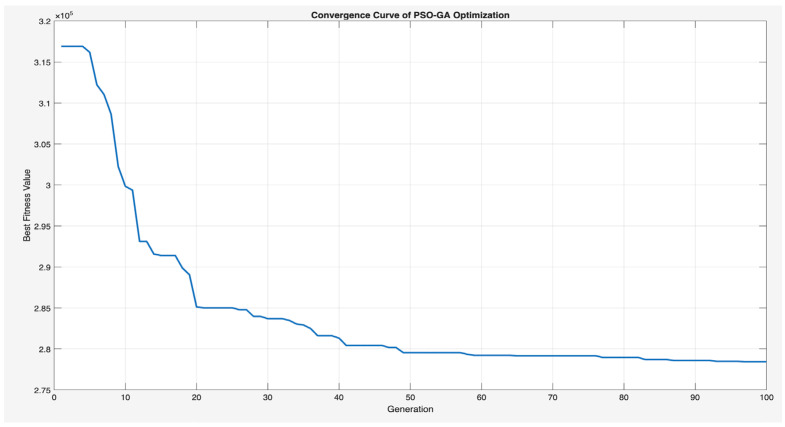
Convergence curve of the hybrid GA-PSO optimization.

Figure 15 illustrates the convergence behavior of the proposed hybrid GA-PSO algorithm, demonstrating a steady decrease in the best fitness value over 100 generations. A fast decline is observed in the earlier iterations, indicating effective global exploration, which is followed by steady stability after around 40 generations, reflecting efficient local exploitation and convergence towards an optimal solution. The algorithm stabilizes at a fitness value of 2.8 × 10^5^, validating the hybrid’s robustness. The computational efficiency of the proposed model was empirically assessed using MATLAB file hybrid_ga_pso_controller_placement.m, wherein the optimization process took approximately 1.5 min to execute 100 generations with a population size of 50 on a MacBook Pro (M2, 8-core CPU, 8 GB RAM). This runtime illustrates that the method shows robust scalability and is computationally feasible for large IoT networks. The convergence curve further confirms the hybrid’s balance between exploration and exploitation, ensuring satisfactory results across various optimization goals.

For comparative analyses, the proposed algorithm was evaluated with other algorithms presented in a related work section. The DEWO [13] algorithm utilized 50–150 nodes with a maximum of 40 controllers, results demonstrating latency and link-failure reductions of 7–20% in comparison with PSO, whereas the proposed approach achieved a 39.6% improvement in latency compared to random placement. The ESFO + POCO [16] algorithm improved latency by 22.2% compared to PSO (about 514 miles or 827 km), but it did not include fault tolerance; in contrast, the proposed model achieved a fault tolerance of 0.60 by overlapping controller coverage. The HSA-PSO [22] lowered latency by 50%, reduced round-trip time from 14.7 ms to 7.3 ms, and achieved a reliability of 0.95, whereas the proposed GA-PSO demonstrates comparable reliability, with a packet loss of 2.35% at a communication range of 3000 m and 74.25% at 1000 m. Overall, the proposed algorithm achieved improved results across multiple metrics, including latency, load balancing, packet loss, redundancy, and fault tolerance, compared to other methods for Kaunas (Lithuania) smart city network, consisting of 2000 nodes and 10 controllers.

## 5. Conclusions

This study presents a hybrid optimization algorithm combining GA and PSO for effective controller placement in large-scale IoT networks. The method was evaluated using real map and geographic data for the city of Kaunas (Lithuania). The proposed approach was evaluated using MATLAB and tested under three scenarios: normal operation, node failures, and traffic spikes. These results were compared against K-Means and random controller placement strategies across multiple performance metrics. The proposed method outperformed the other approaches in all metrics except packet loss and energy use metrics at a communication range of 3000 m, where K-Means achieved 1.40% and 1201.89 (Mbps.m^2^) (×$10^{6})$. The hybrid GA-PSO approach achieved a low average latency of 1459.67 m, improved load balancing with a variance of 45.89 Mbps^2^, and achieved a fault tolerance value of 0.60 at 3000 m communication range. These results indicate that the hybrid algorithm performed well in terms of latency, reliability, and scalability for optimized controller placement in large-scale IoT-based in Kaunas (Lithuania) city. The communication range was found to affect only packet loss and fault-tolerance metrics. These results demonstrate that the proposed method provides a resilient, balanced, and scalable controller placement strategy, which makes it highly suitable for smart city IoT deployments. The proposed hybrid GA-PSO approach exhibits superior performance in large-scale IoT networks compared to random and K-Means; however, the study has certain limitations that must be acknowledged. The analyses relied solely on simulation-based studies instead of implementation in a real-world environment. The network topology was specifically implemented for the city of Kaunas (Lithuania), using its geographic topology and node-density assumptions. This is a realistic case study; however, the outcomes may vary for other cities with different sizes and densities. Additionally, the parameters of the hybrid GA-PSO method (population size, iterations, and weighting factors) were empirically selected and may need modification for different contexts or larger-scale implementations. Future work should address these limitations by implementing the algorithm within a practical SDN-based IoT network, using adaptive traffic models, and validating this methodology across many heterogeneous smart city topologies to ensure generalizability and scalability. In addition, while this study compared the proposed algorithm with random and K-Means placements, future results will include comparisons with metaheuristic algorithms, such as Grasshopper Growth Optimization (GGO) and Takagi–Sugeno Fuzzy Inference System–based Grey Wolf Optimizer (TSFIS-GWO), to further evaluate the performance of the proposed algorithm.

## Figures and Tables

**Table 1:** Comparison of performance improvements: DEWO vs. PSO and FFA.

Topology	Improvement Over PSO	Improvement Over FFA
TataNld	7.82%	2.35%
Deutsche	20.25%	3.55%

**Table 2:** Average CPU Utilization Improvement of MDCLB vs. other algorithms.

Algorithm	Average Improvement Over MDCLB
DLBNB	5%
ESMLB	3%

**Table 3:** Average latency (in miles) for 2 controllers across different methods.

Algorithm	Average Latency (Miles)	Improvement Over Proposed Algorithm
Proposed	400	–
PSO	514	22.2%
Hybrid SD	1733	76.9%
PASIN	4944	91.89%

**Table 4:** Execution Time.

Network Size	Execution Time (Seconds)
100	13
200	36
300	79
400	158

**Table 5:** IoT Traffic generated by a smart neighborhood.

Application	Bandwidth (Mbps)	Latency (ms)	Device Density (/Km^2^)
Autonomous Traffic	0.05–10	10	12,000
Road Safety	0.005	10–100	3000
City Surveillance	20–100	10	60
Structural Health	50–100	1–20	>60,000
Home Energy	0.001–0.1	200–300	6000
Smart Grids	0.001–1.5	1–20	6000
Connected Ambulance	1000	10	60
Remote Monitoring	5	250	60,000

**Table 6:** Execution Time Comparison.

Problem Scale	PACSA-MSCP Time	CPLEX Time
Small-scale	4.2 min	2 h
Large-scale	29.2 min	10 h

**Table 7:** Comparison of Controller placement strategies.

	Algorithm	Metrics Considered	Strengths	Limitations
2.1	DEWO (Hybrid Differential Evolution + Whale Optimization)	Latency, Fault tolerance, Link failure minimization	Improved QoS, Reduced link failure	Clustering imbalance, Static simulation
2.2	GWOAP (Grey Wolf Optimization + Affinity Propagation)	Load distribution, Communication cost	Effective load distribution	No real-world validation, Ignores energy and latency metrics
2.3	MDCLB (Multiple Distributed Controller Load Balancing)	Load balance	Reduced packet loss, minimized response time, Reduced control overhead	Static load scenario, Ignores latency and scalability
2.4	ESFO + POCO (Enhanced Sunflower Optimization + POCO Tool)	Latency, load balancing	Significant latency reduction	Simulation only, Limited scalability tested
2.5	Submodularity-Based Optimization	Execution time, Controller count, Latency	Better execution time	Not validated in dynamic or real-time scenarios
2.6	IVP (IoT-aware VNF Placement)	Latency	Adapts to both static and dynamic traffic	Complexity
2.7	PACSA-MSCP (Ant Colony + Simulated Annealing)	Execution time, deployment cost	Fast execution, Lower deployment cost	Increased algorithmic complexity, Sensitive to node placement
2.8	PHCPA (Partitioned Hybrid Controller Placement Algorithm)	Latency	Fast execution and convergence	Ignores scalability, Fault tolerance
2.9	MOMPA (Multi-Objective Marine Predator Algorithm)	Latency, Load balance	Improve network performance	Computational complexity, Ignores fault tolerance
2.10	HSA-PSO (Hybrid Harmony Search Algorithm + Particle Swarm Optimization)	Propagation delay, Round Trip Time (RTT), Reliability	Reduces propagation delay, improved RTT, increases reliability	Only simulation based; lacks validation under dynamic or real-time network conditions

**Table 8:** Comparison of controller placement strategies for comm. range of 1000 m and 3000 m.

Metric	Optimized		Random		K-Means	
	1000 m	3000 m	1000 m	3000 m	1000 m	3000 m
	Same Result		Same Result		Same Result	
Latency (m)	1459.67		2417.74		1461.49	
Load Variance (Mbps^2^)	45.89		773.61		78.63	
Packet Loss (%)	74.25	2.35	82.50	28.40	76.10	1.40
Energy Use (Mbps.m^2^) (×$10^{6}$)	1265.52		4053.33		1201.89	
Scalability (1/m)	0.000155		0.000139		0.000152	
Redundancy (m)	2937.16		3406.09		2984.81	
Fault Tolerance (fraction 0–1)	0.00	0.60	0.023	0.48	0.00	0.58

**Table 9:** Per-controller node coverage comparison for optimized, random, and K-Means placements under different comm. range.

Controller No:	Optimized (1000 m)	Random (1000 m)	K-Means (1000 m)	Optimized (3000 m)	Random (3000 m)	K-Means (3000 m)
1	70	29	9	209	123	136
2	44	20	38	203	62	179
3	35	61	51	191	257	243
4	49	49	34	186	113	173
5	85	18	36	237	109	167
6	39	25	38	167	177	176
7	39	33	47	173	126	190
8	32	31	75	162	171	238
9	40	40	64	173	225	211
10	82	42	86	252	69	259
Covered Nodes	515	348	478	1953	1432	1972
Uncovered Nodes	1485	1652	1522	47	568	28
Coverage (%)	25.75	17.4	23.9	97.65	71.6	98.6

## Data Availability

The data presented in this study are restricted.
